# Proenkephalin A 119–159 in Perioperative and Intensive Care—A Promising Biomarker or Merely Another Option?

**DOI:** 10.3390/diagnostics14212364

**Published:** 2024-10-23

**Authors:** Paulina Walczak-Wieteska, Konrad Zuzda, Jolanta Małyszko, Paweł Andruszkiewicz

**Affiliations:** 1 2nd Department of Anaesthesiology and Intensive Care, Medical University of Warsaw, 02-097 Warsaw, Poland; paulina.walczak-wieteska@wum.edu.pl (P.W.-W.); pawel.andruszkiewicz@wum.edu.pl (P.A.); 2 Department of Nephrology, Dialysis and Internal Medicine, Medical University of Warsaw, 02-091 Warsaw, Poland; jolanta.malyszko@wum.edu.pl; 3 Doctoral School, Medical University of Warsaw, 02-091 Warsaw, Poland

**Keywords:** acute kidney injury, biomarker, proenkephalin

## Abstract

Acute kidney injury (AKI) is a severe and prevalent syndrome, primarily observed in intensive care units (ICUs) and perioperative settings. The discovery of a new biomarker for kidney function and injury, capable of overcoming the limitations of traditional markers, has the potential to improve the diagnosis and management of AKI. Proenkephalin A 119–159 (PENK) has emerged as a novel biomarker for AKI and has been validated in various clinical settings. It has demonstrated a faster response to AKI compared to creatinine and has been shown to predict successful weaning from renal replacement therapy in the ICU. PENK has also shown promise as an AKI biomarker in perioperative patients. Additionally, PENK has been proven to be effective in estimating mortality and morbidity in patients undergoing cardiac surgery, and those with traumatic brain injury or ischemic stroke. Incorporating PENK into a novel estimation of the glomerular filtration rate, referred to as the PENK-Crea equation, has yielded promising results.

## 1. Introduction

### Molecular and Physiological Background

Acute kidney injury (AKI) is a common clinical syndrome in perioperative care and intensive care units (ICUs). Its development is associated with increased morbidity and mortality, as well as a risk of progression to chronic kidney disease (CKD) [1]. Efforts to identify a highly sensitive and specific early biomarker for AKI detection have been ongoing for over two decades [2]. The gold standard for AKI diagnosis is based on decreased urine output (UOP) and elevated serum creatinine (SCr) levels. However, this approach has significant limitations, including delayed diagnosis and insufficient specificity. SCr levels can be influenced by fluctuating muscle mass, medication changes, diet, cachexia, and hydration status [3]. An increase in SCr indicates that nephron damage has already occurred and that the glomerular filtration rate (GFR) is significantly impaired [4]. Therefore, SCr levels do not accurately reflect current renal function, especially in cases of rapidly developing multi-organ failure or sepsis. Other biomarkers, such as Neutrophil Gelatinase-Associated Lipocalin (NGAL) and cystatin C (CysC), can detect AKI earlier but also have considerable limitations. CysC can detect AKI up to two days before an increase in SCr [5,6], but its levels can be affected by age, gender, race, and inflammation [7]. NGAL levels rise 24–48 h before SCr levels increase. However, significant extrarenal secretion of NGAL may occur due to pre-existing CKD or systemic stress [8,9].

Proenkephalin (PENK) is a novel biomarker for kidney function and injury, serving as a stable surrogate for endogenous opioids known as enkephalins [10]. The terminology for the peptide “proenkephalin amino acid 119–159” varies in the literature, with terms such as proenkephalin A 119–159, proenkephalin, pro-ENK, pENK, or PENK used interchangeably [10,11,12]. This review will standardize our references, using PENK for the peptide, preproenkephalin A for the precursor molecule, and the PENK gene for the corresponding gene.

Preproenkephalin A is a 267-amino acid peptide translated from the PENK gene. It is a precursor for the enkephalin family and cleaves into smaller peptides, including met-enkephalin, leu-enkephalin, and PENK [12]. Enkephalins function as endogenous opioids that interact with delta opioid receptors [13]. The largest number of these receptors are located in the central nervous system, followed by the kidneys [14]. The role and function of enkephalins in the kidneys are not fully understood, with possible regulation of diuresis and natriuresis [15]. While enkephalins have a short half-life of about 15 min, PENK is stable for 48 h, without sex or age impacting on its level [16]. PENK is filtrated by the glomeruli and is not bound by plasma proteins, making it a more practical compound for studying in routine practice [11,16].

## 2. PENK as a Functional Biomarker

A functional biomarker that accurately reflects the GFR and assesses kidney function should possess the following characteristics [17]. It should be predominantly eliminated from the bloodstream through glomerular filtration in the kidneys, with its levels primarily dependent on the kidneys’ filtration capacity. After filtration, the ideal biomarker should not undergo significant secretion or reabsorption in the kidney tubules. This ensures that its concentration reflects glomerular filtration rather than tubular function. Furthermore, the biomarker’s production and clearance should be independent of factors such as muscle mass, hydration status, or nutritional intake, which is essential to avoid inaccurate GFR estimations, particularly in critically ill patients. The biomarker should consistently perform well across diverse patient groups, including those with both stable and impaired kidney function. PENK has been proposed as a superior alternative to other biomarkers due to its glomerular filtration and minimal tubular influence [18].

From the international multicenter study by Beunders et al. [4] was developed a new equation to estimate the glomerular filtration rate (eGFR) using PENK, called the PENK-Crea equation. The study found that the PENK-Crea equation outperformed the Modification of Diet in Renal Disease (MDRD) and 2009 Chronic Kidney Disease Epidemiology Collaboration (CKD-EPI) equations in estimating the GFR. The PENK-Crea equation performed similarly to the 2021 CKD-EPI equation. The study included 1354 patients with both stable and unstable kidney function, with the actual GFR measured using the iohexol or iothalamate clearance methods. The researchers also concluded that adding sex and race variables to the PENK-Crea equation did not improve its accuracy.

## 3. PENK in AKI

### 3.1. Prediction of AKI

Multiple studies have identified PENK as an early indicator of AKI in various patient populations. A 2023 meta-analysis [19] concluded that PENK shows promise as a biomarker for the early prediction of AKI, with a cutoff value of 57.3 pmol/L. Furthermore, analyses revealed that patients with lower PENK levels were at a significantly reduced risk of developing AKI. The authors also noted that results from two studies suggest that serial PENK measurements, rather than a single baseline measurement, may serve as a more reliable indicator of AKI development. However, it is important to note that the studies included in this meta-analysis were limited in number and involved different patient cohorts (septic, postoperative, and CKD). The effectiveness of PENK as an early biomarker for AKI varied among these groups. Using PENK as an early indicator of AKI may lead to a higher rate of false positive results in populations with heart failure and hypertension, and it may not be the most optimal biomarker for these groups. Additionally, it is worth mentioning that different methods of PENK analysis were used across the studies. Nine studies used immunoluminometric assays, one employed an enzyme-linked immunosorbent assay (ELISA), and one did not specify the method used (Table 1).

### 3.2. PENK in Septic AKI

The accuracy of diagnosing AKI in sepsis based on SCr is significantly reduced due to decreased creatinine production and increased blood volume resulting from intensive fluid therapy. Two studies [4,20] involving over 1000 ICU patients in septic shock demonstrated that serum PENK measurement is a reliable indicator of the actual GFR. This has been validated by comparing the PENK levels to iohexol plasma clearance, a more precise and reliable method of measuring the GFR than SCr-based assessments alone. As a result of these studies, a new eGFR equation based on PENK was developed and validated. PENK proved to be more accurate and provided results faster than the previously used methods. This was confirmed by a large, prospective, international study, *The Kidney in Sepsis and Septic Shock* (Kid-SSS) [12]. The Kid-SSS study was conducted across 24 centers in five European countries to investigate the association between PENK levels, AKI, and worsening renal function (WRF) in ICU patients with severe sepsis or septic shock. The study concluded that the PENK levels upon admission correlated with the occurrence of AKI and WRF in septic patients. PENK was significantly elevated in the patients with persistent AKI (53 pmol/L vs. 133 pmol/L) and WRF (70 pmol/L vs. 174 pmol/L) after adjusting for confounding factors. An increase in the PENK levels preceded the elevation of serum creatinine in WRF and was associated with lower levels during renal recovery.

Another single-center observational study conducted on 644 septic patients [21] demonstrated that a single measurement of PENK had predictive value for AKI. However, the association between PENK and AKI was not independent of the eGFR at admission, which was expected, as SCr, by definition, contributes to the AKI endpoint. The study also found that PENK was a better predictor of the 28-day mortality compared to eGFR and was independently associated with the severity of multiple organ failure (MOF).

The results from the ALBIOS multicenter randomized trial [22], involving 956 patients with severe sepsis or septic shock admitted to Italian ICUs, showed that the serum PENK levels were proportional to the severity of renal dysfunction and independently predicted short-term incident AKI. Another study investigated the utility of PENK in identifying septic patients with subclinical AKI (sAKI). A prospective observational study by Dépret et al. [23] measured the PENK levels upon ICU admission and found that 11.7% to 17.5% of the patients had sAKI, defined as a PENK level above 80 pmol/L in patients not meeting the AKI criteria based on the Kidney Disease: Improving Global Outcomes (KDIGO) guidelines. An important finding was that mortality in the patients with sAKI was higher than in those without sAKI and similar to that of the patients meeting the KDIGO criteria for AKI. PENK thus enabled the identification of subpopulations with an intermediate risk of death.

### 3.3. Contrast-Induced AKI

Contrast-induced AKI (CI-AKI) is characterized by a decline in kidney function following the administration of contrast media, in the absence of other conditions that could explain kidney failure [24]. CKD is a major risk factor for CI-AKI development [36]. Given that the pathophysiology of CI-AKI remains poorly understood [37] and there is no consensus on preventive strategies, early biomarker detection would be highly beneficial. The efficacy of PENK measurement for early AKI diagnosis was investigated in a multicenter randomized trial [24] involving CKD patients undergoing contrast-enhanced radiological procedures. The study’s results revealed that the baseline PENK levels before contrast administration were significantly correlated with stable kidney function post-procedure. However, the baseline PENK and SCr levels were similar between the patients with and without CI-AKI and did not predict subsequent CI-AKI. In serial measurements, the PENK levels increased significantly after contrast administration in patients who developed CI-AKI (also termed creatinine-blind AKI). It has been suggested that monitoring PENK over time may be more effective in detecting CI-AKI compared to repeated SCr measurements. Changes in PENK levels (ΔPENK) may enable the earlier detection of AKI that cannot be identified through SCr alone. ΔPENK has been shown to accurately detect creatinine-blind AKI up to 24 h before changes in SCr are observable.

### 3.4. PENK in Perioperative Non-Cardiac Surgery Setting

Several studies have explored the clinical application of PENK in predicting AKI among patients undergoing non-cardiac surgery. While studies on septic patients typically involve large cohorts, those focusing on perioperative patients tend to be smaller and more homogeneous, often including fewer than 100 participants and concentrating on specific surgical procedures. Most research has focused on open and endovascular aortic aneurysm repair (EVAR). Intraoperative stent graft implantation in the aorta with branches to the renal arteries, superior mesenteric artery, and celiac trunk may induce temporary impairment of renal perfusion. Patient comorbidities, such as CKD, severe atherosclerosis, or insufficient cardiovascular reserve, have also been identified as risk factors for AKI following EVAR [38]. Novel biomarkers like PENK may aid in identifying imminent kidney function decline and guide the initiation of targeted therapy. Doukas et al. [25], in a prospective observational study of patients undergoing open thoracoabdominal aortic aneurysm (TAAA) repair, found a strong correlation between elevated PENK levels and AKI requiring renal replacement therapy (RRT) within 12 h postoperatively.

A similar significant correlation between preoperative PENK levels and AKI was found in the study by Gombert et al. [26], which examined 33 patients before and after elective open or endovascular TAAA repair. PENK significantly correlated with AKI occurrence within 48 h postoperatively. In a pilot study conducted by Walczak-Wieteska et al. [27] on 34 patients undergoing elective branched EVAR (BEVAR), the PENK levels were found to correlate with the SCr levels. However, no earlier perioperative increase in PENK levels relative to creatinine was observed.

Lima et al. [28] conducted a study on 57 patients who underwent liver transplant procedures. They found that PENK was an independent predictor of severe AKI and detected AKI 48 h earlier than SCr.

Although studies support the efficacy of PENK as a biomarker for perioperative AKI, discrepancies exist regarding whether the PENK levels increase earlier than creatinine levels in the perioperative period. Given that all these studies involved small patient cohorts, it seems prudent to increase the sample size and broaden the range of surgical procedures studied.

### 3.5. PENK in Cardiac Surgery Setting

A new application of PENK has been evaluated in cardiac surgery patients at high risk of postoperative shock and subsequent AKI, requiring renal replacement therapy (RRT) and resulting in prolonged ICU stays. To enhance the current formula for estimating the risk of perioperative complications, the European System for Cardiac Operative Risk Evaluation II (EuroSCORE II) was augmented with PENK as a predictive biomarker. Hall et al. [29] demonstrated that incorporating PENK into EuroSCORE II to predict an ICU length of stay (ICU LOS) >24 h and ICU LOS >24 h plus mortality yielded statistically significant results, with measurements taken at the end of cardiopulmonary bypass (CPB) and 24 h postoperatively. The highest Area Under the Curve (AUC) was observed when combining EuroSCORE II with PENK measured 24 h after surgery, indicating significant additional value (*p* < 0.05). This study, along with two others on cardiac surgery patients, reported significantly higher preoperative PENK levels in patients who developed perioperative AKI compared to those who did not [29,30,39]. Moreover, the PENK levels were found to correlate with pre-existing CKD, the incidence and severity of AKI, and the requirement for RRT [29,30]. Notably, several studies observed a decrease in the circulating PENK levels over time during and after open-heart surgery, likely due to changes in volume status, intraoperative cardiac arrest, and hypothermia. Despite the promising results, further study and validation of the modified EuroSCORE II are needed due to the small sample sizes. PENK has shown potential as a predictor of mortality and has added significant prognostic value to EuroSCORE II for risk stratification.

### 3.6. PENK in Liberation from Renal Replacement Therapy in Critically Ill Patients

RRT is a common and necessary intervention in critically ill patients with AKI. Assessing kidney function during continuous dialysis is challenging when relying solely on laboratory parameters, as the only available measure is urine output, which has low sensitivity and lacks robust validation studies [40]. A post hoc analysis of the ELAINE monocentric trial, which included 210 ICU patients, primarily those undergoing cardiac surgery [31], measured the PENK levels three days after the initiation of RRT. The analysis demonstrated that PENK could predict early and successful weaning from RRT in critically ill patients with AKI. To validate these findings, data and plasma samples from the randomized controlled multicenter RICH trial were used [32]. This trial enrolled 596 critically ill patients with AKI requiring continuous RRT. Blood samples were collected before RRT initiation and on the third day of therapy. Patients with low pre-RRT PENK levels at the start of RRT were more likely to be successfully weaned off RRT earlier compared to those with high pre-RRT PENK levels. Additionally, low PENK concentrations on the third day were associated with the successful discontinuation of RRT. These findings align with the authors’ hypothesis that low PENK levels indicate preserved baseline renal function, even in patients meeting KDIGO criteria for AKI. Furthermore, the analysis revealed that among patients who received early versus late RRT, those with high pre-RRT PENK levels were more likely to be successfully weaned off RRT sooner when they were in the “early RRT initiation” group. The results of this randomized trial suggest that proenkephalin may be a valuable tool for identifying patients who could benefit from early RRT.

### 3.7. PENK in Prediction of Mortality and Morbidity

The mortality risk at ICU admission is evaluated using simplified scoring systems. One such scale, the Acute Physiology and Chronic Health Evaluation 3 (SAPS-3), is based on classic laboratory measurements, physiological parameters, and comorbidities, and has been validated as a reliable prognostic model for clinical use [41]. Modern biomarkers could be integrated into this scale or used as independent predictors of mortality and morbidity. Several studies have assessed the utility of PENK in this context [42]. These studies have included patients from various cohorts, not limited to those with AKI, and have leveraged the biomarker’s tissue specificity, as proenkephalin receptors are found in the nervous system, blood vessels, and kidneys. For instance, the concentration of PENK in cerebrospinal fluid is 100 times higher than in serum [10]. PENK has been shown to be elevated in ischemic stroke, correlating with the severity of brain injury, and may predict mortality in the acute phase of ischemic stroke [43]. Moreover, PENK has been identified as an independent prognostic marker for both early and six-month mortality after severe traumatic brain injury [44]. In a mixed ICU population, including patients with sepsis, cardiac arrest, and trauma, the PENK levels measured at admission correlated with the 30-day mortality and neurological dysfunction [23]. Elevated PENK levels at ICU discharge were also associated with poor one-year outcomes, even in patients with normal serum creatinine levels at discharge [45].

Among septic patients, elevated PENK levels were correlated with cardiovascular failure. Furthermore, the PENK levels were associated with renal dysfunction on the second day, as measured by the renal component of the Sequential Organ Failure Assessment (SOFA) Score [33]. A study examining mortality in patients who did not meet the KDIGO criteria for AKI revealed additional findings. Research investigating the association between PENK and the incidence of subclinical AKI (sAKI) in septic ICU patients [23] found that patients with sAKI had similar mortality rates to those without AKI (Table 2).

## 4. PENK in Children

A 2020 study [46] examined the utility of PENK as a biomarker for AKI in children. The authors established reference values for PENK in healthy children under one year of age. They then compared the PENK levels in critically ill infants with and without AKI and assessed PENK’s performance in relation to the established AKI biomarkers CysC and β-trace protein (BTP). The study found that the PENK levels in 100 healthy infants remained stable during their first year of life, with a median level of 517.3 pmol/L, which was significantly higher than the PENK levels observed in adults. In 91 critically ill infants, the median PENK levels were significantly higher in those with AKI compared to those without. Furthermore, PENK demonstrated a potentially greater accuracy than CysC and BTP in diagnosing AKI within the first 24 h after intubation. The authors concluded that PENK is a promising biomarker for pediatric AKI, emphasizing the necessity of age-normalized reference values for accurate diagnosis. The study by Smeets et al. [47], which included 97 patients aged 0–18 years, found that the PENK levels were age-dependent, reaching adult values around two years of age. Additionally, PENK outperformed SCr and CysC in correlating with GFR, particularly in children older than one month. While a PENK-based eGFR equation for children is not yet available, the study suggests its potential for more accurate GFR estimation and AKI diagnosis in children.

## 5. PENK Combined with Other AKI Biomarkers

Jäntti et al. [34] measured the plasma PENK and NGAL levels in 154 patients with cardiogenic shock as part of the prospective CardShock study. High levels of PENK and NGAL at baseline were independently associated with AKI. Moreover, elevated levels of PENK and NGAL at 24 h were strong and independent predictors of the 90-day mortality.

The study by Gayat et al. [48] compared PENK with a combination of Tissue Inhibitor of Metalloproteinase 2 and Insulin-like Growth Factor-binding Protein 7 (TIMP2xIGFBP7) to predict AKI in ICU settings. Both PENK and TIMP2xIGFBP7 were measured upon ICU admission in the plasma and urine. It was found that the PENK and TIMP2xIGFBP7 levels at admission correlated with the severity of AKI. However, PENK, as a filtration marker, demonstrated a significantly stronger correlation with AKI. Elevated PENK levels predicted the need for RRT more accurately than elevated TIMP2xIGFBP7 levels. Liu et al. [35] prospectively evaluated the predictive value of the plasma PENK and adrenomedullin levels for septic AKI, comparing them to other candidate biomarkers such as NGAL, CysC, kidney injury molecule-1, and interleukin-18 in 42 septic patients in the ICU. The study revealed a significant predictive value for PENK and adrenomedullin in predicting AKI in septic patients.

sAKI is common among ICU patients. Stage 1S AKI has been proposed as part of a new definition to reflect a subclinical syndrome, characterized by elevated biomarkers without a rise in SCr [49]. sAKI is associated with poor outcomes, including mortality rates comparable to those of clinically evident AKI. An analysis conducted by Boutin et al. of the urinary peptides NGAL, CysC, PENK, and liver fatty acid-binding protein (LFABP) in an extensive study of 1154 patients revealed similar pathological pathways in both sAKI and AKI, primarily involving inflammation, hemolysis, and endothelial dysfunction [50]. Notably, the presence of multiple positive biomarkers in patients without AKI was strongly correlated with an increased risk of death within one year. The study suggested that combining kidney biomarkers could enhance the early diagnosis and treatment of AKI, potentially improving patient outcomes.

## 6. Conclusions

Numerous studies focused on perioperative and critically ill cohorts of patients have demonstrated that PENK is a useful and precise functional biomarker of AKI. Sequential PENK assays provide the most accurate results and offer high sensitivity in detecting AKI. Additionally, PENK has been proven to be helpful in the prediction of patient mortality. Finally, due to its role as a biomarker for glomerular filtration, it could also be utilized to calculate a new eGFR formula.

## Figures and Tables

**Table 1:** Overview of studies evaluating the application of PENK in the AKI diagnosis.

Author	Patients (*n*)	Population	Males	Age [Median]	AKI [%]	PENK ^4^
Beunders et al. [4]	1354	mixed	59%	48–68	N/A	once
Marino et al. [11]	101	ED ^3^	61%	78	49%	once
Hollinger et al. [12]	583	ICU	63%	66	62%	once
Beunders et al. [20]	23	ICU ^1^	65%	63	52%	multiple
Rosenqvist et al. [21]	588	ICU	51%	73	16%	once
Caironi et al. [22]	895	ICU	58%	70	28%	multiple
Depret et al. [23]	2004	ICU	65%	63	47%	once
Depret et al. [23]	583	ICU	62%	66	62%	once
Breidthardt et al. [24]	111	CKD ^2^	69%	77	7%	multiple
Doukas et al. [25]	36	elective surgery	69%	56	33%	multiple
Gombert et al. [26]	33	elective surgery	52%	63	52%	multiple
Walczak-Wieteska et al. [27]	34	elective surgery	71%	78	35%	multiple
Lima et al. [28]	57	elective surgery	61%	58	88%	multiple
Hill et al. [29]	136	elective surgery	64%	66	13%	multiple
Mossanen et al. [30]	107	elective surgery	77%	69	20%	multiple
van Groote et al. [31]	369	ICU	67%	69	100%	multiple
van Groote et al. [32]	210	ICU	63%	70	100%	multiple
Frigyesi et al. [33]	1978	ICU	61%	66	N/A ^5^	multiple
Jantti et al. [34]	154	ED	74%	66	31%	multiple
Liu et al. [35]	42	ICU	N/A	N/A	38%	once

^1^ ICU—intensive care unit, ^2^ CKD—chronic kidney disease, ^3^ ED—emergency department, ^4^ PENK—Proenkephalin A 119–159 measurement, ^5^ Not-applicable.

**Table 2:** Summary of PENK concentrations across various clinical settings and outcomes.

Authors	Setting	PENK (pmol/L)	Outcome	Type ^4^
Doukas et al. [25]	Surgical	93.9	AKI in first 12 h	Observed
Walczak-Wieteska et al. [27]	Surgical	140.95	AKI in first 24 h	Observed
Lima et al. [28]	Surgical	90.16	Mild AKI	Observed
Mossanen et al. [30]	Surgical	96	AKI	Observed
Liu et al. [35]	ICU ^1^	229.2	Septic AKI	Observed
Frigyesi et al. [33]	ICU	103	Sepsis	Observed
von Groote et al. [31]	ICU	89	Liberation from RRT	Calculated
von Groote et al. [32]	ICU	89	Liberation from RRT	Calculated
Hollinger et al. [12]	ICU	84.1	Increased mortality	Calculated
Jäntti et al. [34]	ED ^2^	84.4	Increased mortality	Calculated
Liu et al. [35]	ICU	66.97	Prediction of AKI	Calculated
Rosenqvist et al. [21]	ICU	>100	Renal events	Defined
Dépret et al. [23]	ICU	>80	sAKI	Defined
Breidthardt et al. [24]	CKD ^3^	>80	sAKI	Defined

^1^ ICU—intensive care unit, ^2^ ED—emergency department, ^3^ CKD—chronic kidney disease, ^4^ Type. Observed as cumulative median. Calculated value to describe predictive indicator. Defined PENK concentration before the study.

## Data Availability

No new data were created or analyzed in this study.
